# Editorial: Nontuberculous mycobacterial pulmonary disease: immunopathogenesis and immunological risk factors

**DOI:** 10.3389/fimmu.2025.1582489

**Published:** 2025-03-21

**Authors:** Edward D. Chan, Veronica Schmitz

**Affiliations:** ^1^ Department of Academic Affairs, National Jewish Health, Denver, CO, United States; ^2^ Division of Pulmonary Sciences and Critical Care Medicine, University of Colorado Anschutz Medical Campus, Aurora, CO, United States; ^3^ Department of Medicine, Rocky Mountain Regional Veterans Affairs Medical Center, Aurora, CO, United States; ^4^ Laboratório de Hanseníase, Instituto Oswaldo Cruz, Fundação Oswaldo Cruz, Rio de Janeiro, Brazil

**Keywords:** NTM (nontuberculous mycobacteria), NTM infections, NTM disease, NTM host interaction, NTM therapy

Mycobacteria are comprised of *Mycobacterium tuberculosis* complex, *Mycobacterium leprae*, and non-tuberculous mycobacteria (NTM). While NTM contain approximately 200 distinct species and subspecies, diseases in humans are predominantly caused by three main groups: *(i) Mycobacterium avium* complex (which is comprised of several members, with the most clinically relevant being *M. avium* subsp *hominissuis*, *M. intracellulare* subsp *intracellulare*, and *M. intracellulare* subsp *chimaera*); *(ii) Mycobacterium abscessus* group (which consists of three subspecies: *abscessus*, *massiliense*, and *bolletii*); and *(iii) Mycobacterium kansasii* complex. NTM are ubiquitous in natural and built environs, especially in fresh water, soil, and biofilms, making NTM infections environmentally- acquired.

Upon encountering a patient with an NTM infection, four elements should be determined: the specific NTM responsible, how the infection was contracted, the location and extent of the infection, and the presence of any host risk factors. The last two elements are interrelated because the degree of underlying host risk factors often dictates the scope of the infection. Thus, it is useful to categorize the NTM infection into one of four main types: *(i)* skin, soft tissue, and osteoarticular infections, which are typically due to accidental trauma or medical procedures; *(ii)* isolated infection of the head and neck lymph nodes (also known as cervical lymphadenitis or “scrofula”); *(iii)* isolated NTM lung disease; *(iv)* disseminated or extrapulmonary visceral disease. Identifying or distinguishing the latter two types helps dictate what investigative actions to embark upon to determine the presence of any underlying host risk factors.

This Research Topic of *Frontiers in Immunology*, titled “*Nontuberculous mycobacterial pulmonary disease: immunopathogenesis and immunological risk factors*,” is comprised of five papers. Hicks et al. investigated the pathogenesis of NTM disease from the host’s perspective. Yang et al. and Wang et al. examined specific interactions of the host with NTM and *M. tuberculosis*, respectively. Keefe et al. focused on how a specific NTM species is able to thrive in the environment and infect host cells. Mediaas et al. investigated a novel host-directed therapy against NTM.

Hicks et al. reported three infants with complete DiGeorge anomaly (cDGA) – a genetic condition resulting in the complete absence of the thymus resulting in severe T cell deficiency – all with disseminated NTM infections. These cases are rare, likely due to the highly infrequent intersection of two uncommon conditions (DGA and NTM infection). The authors recommend that DGA should be considered in the differential diagnoses of children with disseminated NTM infections. Currently, mutations of genes that encode for elements of the interferon-gamma (IFNγ)–interleukin-12 (IL-12) axis should be suspected in very young children with disseminated NTM disease. In adults with disseminated NTM infections, acquired immune disorders like AIDS, anti-IFNγ antibody syndrome, or use of immunosuppressive drugs are more likely risk factors.

Various mouse strains have been used to explore host susceptibility to NTM infections. However, most murine infection models result in systemic NTM infection rather than exhibit isolated NTM lung disease, the latter characterized by bronchiolitis and bronchiectasis in humans. Yang et al. developed a mouse model using intratracheal infection with *M. abscessus* embedded in agar beads, which better simulates airway infection. The infected mice developed granulomas, a common feature *M. abscessus* infection in humans. Others have also employed a fibrin plug model where *M. abscessus* is suspended in thrombin and fibrinogen, trapping the bacteria in the airways as the plugs coagulate (1). Models such as these intuitively more closely replicate isolated NTM lung disease. Future studies could investigate whether such inoculation methods also enhance NTM biofilm formation in the airways, a barrier known to resist penetration by immune cells and antibiotics.

The co-evolution of humans and mycobacteria has enabled reciprocal adaptation in a mutual struggle for dominance. A recurring example is that the same host factors or mycobacterial components can be used by either side to gain an advantage, analogous to a “tug o’ war” event. Wang et al. reviewed the role of CD36, a cell surface scavenger receptor which mycobacteria have hijacked for their own advantage. Although their focus was on *M. tuberculosis*, similar mechanisms may also be involved in the pathogenesis of NTM disease. They discussed that *M. tuberculosis* engages CD36 leading to: *(i)* increased fatty acid uptake by host cells, aiding bacillary persistence; *(ii)* monocyte differentiation into macrophages with an immunosuppressive phenotype; and *(iii)* enhanced uptake of surfactant lipids by macrophages, supporting mycobacterial growth. Additionally, they highlighted studies showing CD36 expression in various cell types that are involved in granuloma formation, including macrophages, granulocytes, lymphocytes, and fibroblasts. It was previously demonstrated that the *M. avium* complex manipulates macrophage lipid metabolism, leading to the formation of lipid-laden foamy macrophages that create a favorable environment for mycobacterial growth (2). Recent studies using a mouse model demonstrated that CD36 expression is elevated on macrophages during *M. avium* infection (3). In line with this, inhibiting CD36 has been shown to reduce the growth of *M. avium*. Choi et al. (4) confirmed that blocking CD36 leads to decreased growth of *M. avium*, which aligns with earlier research. These findings highlight the crucial role of CD36 in the pathology of mycobacterial diseases as well as a potential therapeutic target.

Bronchiectasis, a common feature of chronic NTM lung disease, is characterized by repeated cycles of infection and inflammation. Airway injury may be exacerbated by NTM-generated biofilms, a complex matrix of extracellular polymeric substances – comprised of DNA, proteins, polysaccharides, and lipids – released from live and dead microbes (5–9). NTM lung cavity walls have also been demonstrated to contain biofilms (10). This biofilm matrix provides protection and nutrients for the microbes (11). Keefe et al. identified gene products from *M. abscessus* in biofilms using a transposon library of *M. abscessus* along with surface proteomic analysis. They uncovered specific proteins related to the mycobacteria’s ability to detach and establish new biofilms as well as attach to and infect both epithelial cells and macrophages.

The primary treatment for NTM lung disease involves antibiotics, airway clearance, and targeted therapies for underlying conditions, *e.g.*, anti-retroviral drugs for HIV infection, modulators for defective cystic fibrosis transmembrane conductance regulator, and alpha-1-antitrypsin (AAT) replacement therapy for those with AAT deficiency. For those with disseminated NTM disease, host-directed therapies may include IFNγ replacement therapy for subjects with defects in the IFNγ-IL-12 axis, anti-B cell therapy for those with anti-IFNγ autoantibodies, and immune checkpoint inhibitors for individuals with severe T cell immunosuppression (12, 13), though the lattermost is controversial (13, 14). Adjunct therapies directed more specifically against the mycobacteria include inhibitors of *M. abscessus* β-lactamase for those on β-lactam antibiotics (15), efflux pump inhibitors (16), and biofilm disrupters (8). Mediaas et al. found that metformin, commonly used to treat diabetes mellitus, significantly reduced *M. avium* burden in mice. One mechanism for this salubrious effect was by enhancing the ability of macrophages to kill or control the infection by activating the AMPK (5’ adenosine monophosphate-activated protein kinase) pathway, increasing mitochondrial reactive oxygen species and enhancing phagosome maturation and acidification.

In summary, the five articles featured in this Research Topic enhance our understanding of the pathogenesis of NTM infections. This increase in knowledge is crucial since we are all likely exposed to NTM and yet NTM infections occur in relatively few individuals. The interplay between specific host susceptibility and NTM virulence factors is likely critical in the development of NTM disease. Each of these papers contributes to our collective understanding of this relationship. Ongoing research into innovative strategies and therapies, as highlighted by these five papers, is essential for improving outcomes for patients with NTM disease.
